# P-2295. Risk Factors for and Impact of Enteric Gram-negative Infections on Lung Transplant Clinical Outcomes

**DOI:** 10.1093/ofid/ofae631.2448

**Published:** 2025-01-29

**Authors:** Simran Gupta, R Alfonso Hernandezv Acosta, Elene Chamberlin, Ella Woehl, Erin M Connolly, Audra O’Neill, Antonio Coppolino, Nirmal Sharma, Lindsey R Baden, Ann E Woolley

**Affiliations:** Brigham and Women's Hospital, Chestnut Hill, Massachusetts; Brigham and Women's Hospital, Chestnut Hill, Massachusetts; Brigham and Women's Hospital, Chestnut Hill, Massachusetts; Brigham and Women's Hospital, Chestnut Hill, Massachusetts; Brigham and Women's Hospital, Chestnut Hill, Massachusetts; Brigham and Women's Hospital, Chestnut Hill, Massachusetts; Birigham and Women's Hospital, Boston, Massachusetts; Birigham and Women's Hospital, Boston, Massachusetts; Brigham and Women's Hospital, Chestnut Hill, Massachusetts; Brigham and Women's Hospital, Chestnut Hill, Massachusetts

## Abstract

**Background:**

Bacterial infections such as *Pseudomonas aeruginosa* (PsA) and *S. aureus* lead to increased morbidity post-lung transplant. Less is known about the impact of enteric gram-negative (GNR) infections. This study’s aim was to assess risk factors for developing GNR infections within 6-months of transplant and associated 1-year outcomes compared to lung transplant recipients (LTR) with other bacterial infections or uninfected LTR.

Table 1

**Figure:**
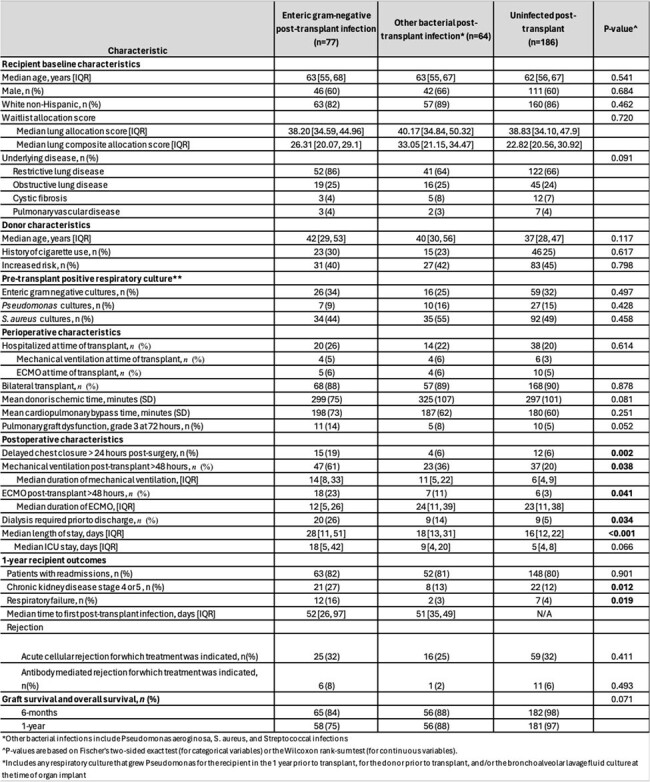

Characteristics and Outcomes of LTR with post-transplant GNR infections, other bacterial infections, and no infection

**Methods:**

A single-center retrospective study of LTR between January 2017 and October 2023 was performed. Donor/recipient characteristics, microbiologic data, and 1-year clinical outcomes were analyzed.

**Results:**

During the study period, 327 patients had a lung transplant with a greater than 30-day survival. 77 (23%) had GNR infections, 64 (20%) had either PsA, *S. aureus*, or Streptococcal infections (infect), and 186 (57%) did not have a bacterial infection within 6-months post-transplant. The GNR infections were: 18% *Enterobacter cloacae complex*, 16% *E.coli*, 25% *Klebsiella sp*., 7% *Acinetobacter sp*., 1% *Serratia sp*., 1% *Citrobacter sp*., 7% *Burkholderia sp*., 19% *Stenotrophomonas sp*., 6% others. Infections included: 19% bacteremia, 91% pneumonia, 5% empyema, 4% mediastinitis, 1% other. Donor, recipient, and perioperative characteristics were similar amongst the cohorts. A similar proportion of LTR donors were colonized with GNR, PsA, and *S. aureus* in the 3 cohorts pre-transplant. A greater proportion of the GNR cohort had delayed chest closure, required dialysis, mechanical ventilation, and/or ECMO for >48 hours, and had a longer ICU and index hospitalization compared to the other cohorts [Table 1]. The median time to infection was similar for the GNR and infect cohorts (52 and 51 days), but a greater proportion of the GNR cohort developed chronic kidney disease or respiratory failure. 6-month and 1-year survival were less in the GNR cohort but not statistically significant.

**Conclusion:**

LTR with more complicated post-operative courses were more likely to develop enteric GNR infections which led to increased post-transplant morbidities. Pre-transplant GNR colonization was not a risk factor for GNR infection post-transplant. Strategies to minimize post-transplant enteric GNR infections need further study.

**Disclosures:**

All Authors: No reported disclosures

